# Helping-Like Behaviour in Mice Towards Conspecifics Constrained Inside Tubes

**DOI:** 10.1038/s41598-019-42290-y

**Published:** 2019-04-09

**Authors:** Hiroshi Ueno, Shunsuke Suemitsu, Shinji Murakami, Naoya Kitamura, Kenta Wani, Yosuke Matsumoto, Motoi Okamoto, Takeshi Ishihara

**Affiliations:** 1 Department of Medical Technology, Kawasaki University of Medical Welfare, Okayama, 701-0193 Japan; 2 Department of Medical Technology, Graduate School of Health Sciences, Okayama University, Okayama, 700-8558 Japan; 3 Department of Psychiatry, Kawasaki Medical School, Kurashiki, 701-0192 Japan; 4 Department of Neuropsychiatry, Graduate School of Medicine, Dentistry and Pharmaceutical Sciences, Okayama University, Okayama, 700-8558 Japan

## Abstract

Prosocial behaviour, including helping behaviour, benefits others. Recently, helping-like behaviour has been observed in rats, but whether it is oriented towards rescue, social contact with others, or other goals remains unclear. Therefore, we investigated whether helping-like behaviour could be observed in mice similar to that in rats. Because mice are social animals widely used in neuroscience, the discovery of helping-like behaviour in mice would be valuable in clarifying the psychological and biological mechanisms underlying pro-sociability. We constrained mice inside tubes. Subject mice were allowed to move freely in cages with tubes containing constrained conspecifics. The subject mice released both cagemates and stranger mice but did not engage in opening empty tubes. Furthermore, the same behaviour was observed under aversive conditions and with anesthetised conspecifics. Interestingly, hungry mice opened the tubes containing food before engaging in tube-opening behaviour to free constrained conspecifics. Mice showed equal preferences for constrained and freely moving conspecifics. We demonstrated for the first time that mice show tube-opening behaviour. Furthermore, we partly clarified the purpose and motivation of this behaviour. An effective mouse model for helping-like behaviour would facilitate research on the mechanisms underlying prosocial behaviour.

## Introduction

Prosocial behaviour comprises actions that benefit others^1^ and is said to include informing, comforting, sharing, and helping^2^. Many animal species have been reported to exhibit prosocial-like behaviour^3,4^. The psychological mechanisms underlying prosocial behaviour are motivated by both selfish and unselfish factors. One of the difficulties here is to establish the psychological basis of prosocial behaviour in young humans before they have attained lingual expertise and in non-human animals^1^. Currently, the psychological basis of prosocial behaviour and its fundamental mechanisms remain unclear^5–8^. Interestingly, in recent years, it has been reported that rats also show prosocial behaviour^9–13^. Rats rescued others in various situations, they donated food, they groomed conspecifics, and they freed trapped conspecifics^9,12^.

Helping behaviour, a form of prosocial behaviour, involves acting for the benefit of others (e.g. rescuing others from difficult situations) in the absence of reward^14^. Ben-Ami Bartal *et al*. constrained rats inside tubes and showed that cagemate rats released the constrained individuals^9–11^. Similarly, Sato *et al*. placed rats in flooded conditions that are considered to be aversive, and cagemate rats released the individuals placed in this condition^12^. They speculated that helping behaviour is based on empathy^9–12^. In contrast, it has been suggested that rats interested in social contact would show helping behaviour towards conspecifics^15–17^. As described above, the motivation and purpose of this helping-like behaviour observed in rats remain undetermined. New experimental methods and models are needed.

In this study, we investigated whether mice, which are also rodents, would show helping-like behaviour, similar to that demonstrated by Ben-Ami *et al*. for rats. It is suggested that the neurobiological systems of prosocial behaviour are shared among mammalian species^18^. Mice are social animals widely used in neuroscience. Establishing prosocial, helping-like behaviour in mice would be of great value in the search for psychological and biological mechanisms that underlie pro-sociability.

In this study, the restraint apparatus of Ben-Ami *et al*. was modified to restrain mice. Confinement inside a tube causes psychological restraint stress without inducing pain to the mouse^19^. The tube restraining the mouse was capped with paper so that it could be easily accessed by the subject mice by eliminating the obstructing paper by chewing or crushing. A mouse cannot open the tube lid by chance. Thus, by investigating the tube-opening behaviour of the mouse in the following five states, we attempted to clarify a part of the psychological mechanism (interest, purpose, and motivation) of tube-opening behaviour in mice:

(1) Testing tube-opening behaviour to free cagemate mice. First, we investigated whether the mouse would show tube-opening behaviour to free cagemate mice constrained inside tubes; (2) Testing tube-opening behaviour to free cagemate and stranger mice. Changes in tube-opening behaviour of subject mice with respect to a cagemate or stranger restrained mouse were compared. Mouse empathy-like behaviour is considered to occur only for cagemate mice^9,20–22^. If this tube-opening behaviour is based on empathy, it was expected that the behaviour would differ depending on whether the restrained conspecific was a cagemate or a stranger; (3) Testing tube-opening behaviour to free anesthetised cagemate mice. We examined if subject mice would show tube-opening behaviour when the cagemate mice in the tube did not move or vocalise. We placed anesthetised cagemate mouse in the tube. If distress signals communicated to the free mouse are required for tube-opening behaviour to occur, the subject mouse was not expected to free an anesthetised mouse. (4) Testing tube-opening behaviour in hungry and non-hungry states. Next, we examined whether subject mice would preferentially open a tube containing food when hungry. We investigated whether tube-opening behaviour changes due to internal factors such as mood and the physical condition of the subject mouse. Moreover, in this experiment, we clarified whether the mouse could identify the contents of the tube. If the mice can identify the tube contents, and if the behaviour is based on self-interest, a difference in the behaviour was expected; (5) Testing tube-opening behaviour under aversive conditions. We examined whether the tube-opening behaviour of the subject mouse would change depending on the external environment. In order to induce discomfort to the mouse, we wet half of the home cage bedding with water and the changes in the tube-opening behaviour of the mouse was examined. In this experiment, we estimated the degree of motivation of the mouse to engage in tube-opening behaviour. We expected that tube-opening behaviour would not be performed under this condition if the motivation was not strong.

## Results

We used the mouse strain C57BL/6N, which is one of the most widely used animal models in biological research.

### Training for opening the paper lid

**Figure 1:**
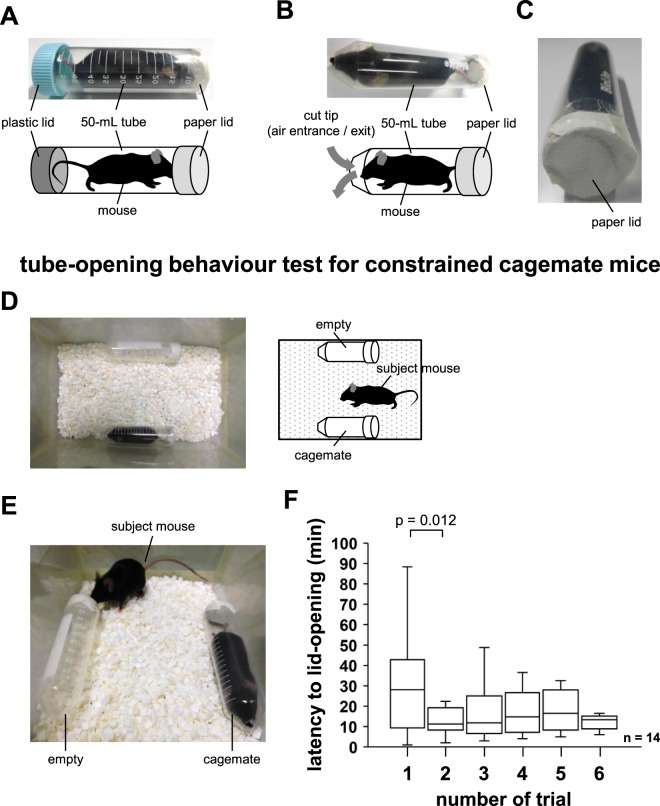
Training for opening the paper lid and tube-opening behaviour test. (**A**) Sample picture and schematic diagram of training for opening the paper lid. One side of the 50-mL tube was closed with a paper lid and the other with a plastic lid. (**B**) Sample picture and schematic diagram of the mouse waiting for release from the 50 mL tube. A 1-cm-diameter hole in front of the mouse and a paper lid at the back. (**C**) A sample image of the tube, containing the mouse, covered at the back with a paper lid. (**D**) Sample picture and schematic diagram of the tube-opening behaviour test in the new home cage. A tube containing the cagemate mouse on one side and an empty tube on the other side. (**E**) Sample picture during the test of tube-opening behaviour. The test mouse freely moves in the cage. (**F**) Tube-opening behaviour test for constrained cagemate: latency to paper lid-opening in each trial. All data are presented as box plots. The p values were calculated uisng one-way repeated measures ANOVA (**F**). n = 14 animals.

Mice were subjected to training for opening the paper lid on being constrained inside 50-mL tubes; the front of the tube was closed with a paper lid and the rear was closed with a plastic lid (Fig. 1A). The tube was placed inside a new home cage, and the mice managed to break through the paper lid and exit the tube. We practiced this exercise three times a day for 2 days. By the end of training, all mice had learned to open the paper lid and exit the tube.

### Mice show tube-opening behaviour to free constrained cagemate mice

In this experiment, we investigated whether mice show tube-opening behaviour to free constrained cagemate mice. The back of the tube containing the mouse was closed with a paper lid (Fig. 1B,C). The subject mouse could release the constrained mouse by gnawing on the paper lid. Constrained cagemate mice were located at the sides of a new home cage. We placed an empty tube on the other side (Fig. 1D). The subject mouse was placed at the centre and allowed to explore the entire home cage for a 90-min session (Fig. 1E). The latency to lid opening of the tube containing the cagemate mouse was measured. We performed the test once a day for 7 days. We observed that subject mice opened the paper lid of tubes containing cagemate mice (Sup. Video 1). Seventy percent of mice learned to open the lid. Mice that did not learn to open the lid were excluded from the analysis. The latency to lid opening was significantly reduced in the second trial (Fig. 1F). In the subsequent trials, the latency remained almost unchanged. Subject mice opened the paper lid of the tube containing the cagemate mouse in all seven trials (Fig. 1F, *F*_6,91_ = 2.3, p = 0.05). Some subject mice bit the lid of the empty tube. However, in all trials, subject mice did not open the paper lid of the empty tube placed on the other side of the cage (Fig. 1D,E). We also observed that subject mice often exhibited tube-opening behaviour and then entered the tube.

### Mice also show tube-opening behaviour to free constrained stranger mice

**Figure 2:**
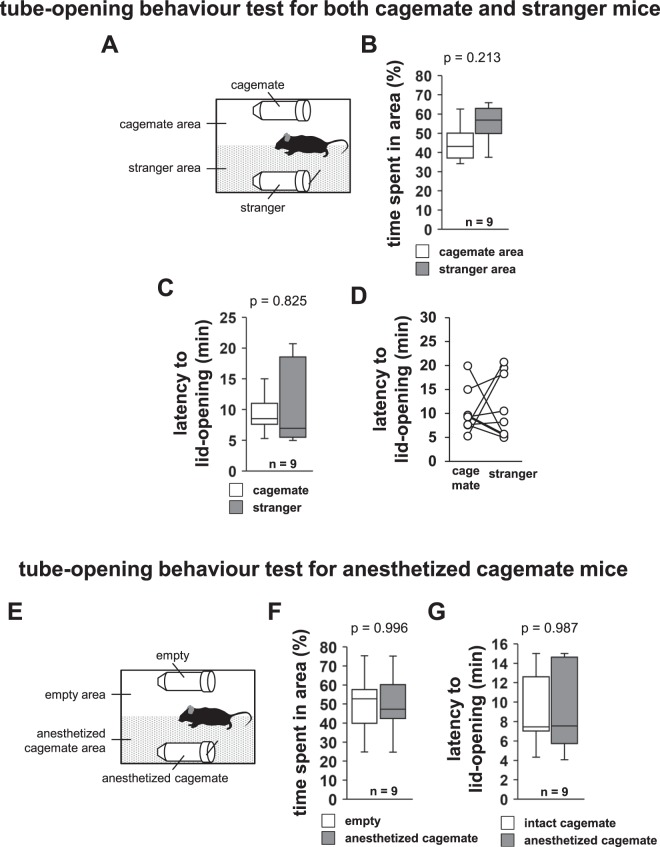
Tube-opening behaviour test for both cagemate and stranger mice, and tube-opening behaviour test for anesthetised cagemate mice. (**A**) Schematic diagram of the test. Tube-opening behaviour test for cagemate and stranger mice: time spent in the area (**B**) and latency to lid-opening (**C**). (**D**) Individual latency to lid-opening in the tube-opening behaviour test for both cagemate and stranger mice. (**E**) Schematic diagram of this test. (**F**) Tube-opening behaviour test for anesthetised cagemate mice: time spent in the anesthetised cagemate area or empty area. (**G**) Comparison of the latency to lid-opening for an anesthetised and a non-anesthetised mouse. All data are presented as box plots. The p values were calculated using paired t-test (**B,F**), Wilcoxon signed-rank test (**C**), and one-way repeated measures ANOVA (**G**). n = 9 animals per test.

Next, we investigated whether the subject mice would show a preference for opening the tube containing a cagemate over that containing a stranger mouse (Fig. 2). In this test, cagemate and unfamiliar C57BL/6N male (stranger) mice that had no previous contact with the subject mouse were placed into one of the transparent tubes located on both sides of a new home cage (Fig. 2A). Subject mice showed no significant differences in the time spent in the two cage areas (Fig. 2B, *t*_16_ = −1.369, p = 0.213). No significant difference was detected between the cagemate and stranger mouse conditions with respect to the latency to lid-opening (Fig. 2C,D, T = 23, n = 9, p = 0.825). Five of the nine mice opened the paper lid of the tube containing the cage mate first. These results suggested that there was no difference in tube-opening behaviour to free cagemate and stranger mice.

### Tube-opening behaviour to free constrained anesthetised cagemate mice

We prevented the constrained mouse from expressing or feeling distress by anesthetizing it. We performed this experiment by placing an anesthetised cagemate in a tube. Subject mice spent almost the same amount of time in both areas (Fig. 2F, *t*_16_ = 0.006, p = 0.996). We observed that subject mice opened the paper lid of the tube containing the anesthetised cagemate (Sup. Video 2). We compared the latency to lid-opening when the cagemate was in an anesthetised and in an un-anesthetised state. Subject mice demonstrated equivalent latencies to lid-opening in both states (Fig. 2G, *F*_1,16_ = 0.0, p = 0.987). These results suggested that the mice exhibited the similar behaviour towards their cagemates irrespective of their state (anesthetised or un-anesthetised).

### Tube-opening behaviour in hungry and non-hungry states

**Figure 3:**
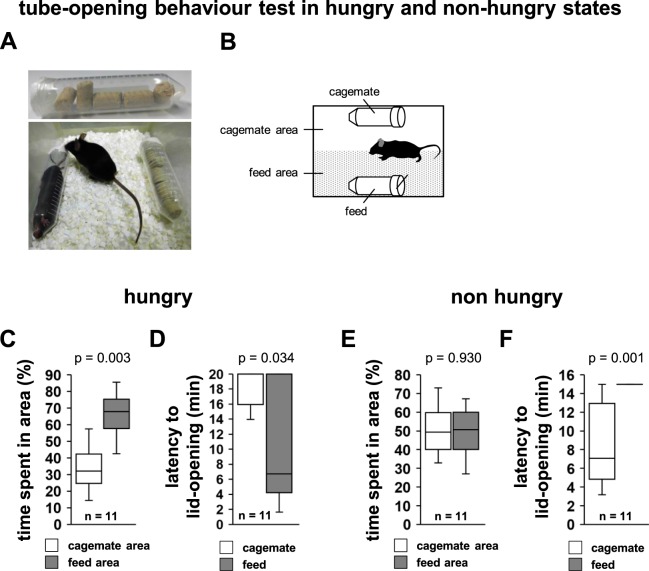
Tube-opening behaviour test in hungry and non-hungry states. (**A**) Upper row: Sample picture of the 50-mL tube containing food. It is closed with a paper lid. Lower row: sample picture during the test of tube-opening behaviour in the hungry state. The tube containing the cagemate is located on the left side and the tube containing the food on the right side. The test mouse moves freely around the cage. (**B**) Schematic diagram of this test. The cagemate area refers to the half of the home cage with the tube containing the cagemate mouse, and the feed area refers to the half of the home cage with the tube containing the food. Tube-opening behaviour test for cagemate versus feed in the food-deprived state: time spent in the area (**C**) and latency to lid-opening (**D**). Tube-opening behaviour test for cagemate versus feed in the non-food deprived state: time spent in the area (**E**) and latency to lid-opening (**F**). All data are presented as box plots. The p values were calculated using paired t-test (**C,E**) and the Mann-Whitney’s *U*-test (**D,F**). n = 11 animals per test.

For this experiment, we used two transparent tubes, one containing food and the other containing a cagemate mouse (Fig. 3A,B). Next, we examined whether subject mice would preferentially open the tube containing the food when in a hungry state. To induce hunger, we restricted access to food from the day before this test. Subject mice spent a significantly longer time in the area with the tube containing the food than in the area with tube containing the cagemate (Fig. 3C, *t*_20_ = −3.975, p = 0.003). We measured the time spent in the area before opening the tube containing the cagemate. Therefore, this result also includes the time that mice spent in eating, near the food tube. The latency to lid-opening was significantly shorter for the tube containing the food than for the one containing the cagemate (Fig. 3D, U = 28, n_1_ = n_2_ = 11, p = 0.034).

Next, we performed the same experiment using subject mice that were not food deprived (Fig. 3E,F). There were no significant differences between the time spent in the area with the tube containing the food and the tube containing the cagemate (Fig. 3E, *t*_20_ = 0.090, p = 0.930). Subject mice that were not in a hungry state did not actively engage in opening the lid of the tube containing the food (Fig. 3F, U = 4, n_1_ = n_2_ = 11, p = 0.001). These results indicate that hungry subject mice prioritise food acquisition over helping constrained conspecifics.

### Tube-opening behaviour under aversive conditions

**Figure 4:**
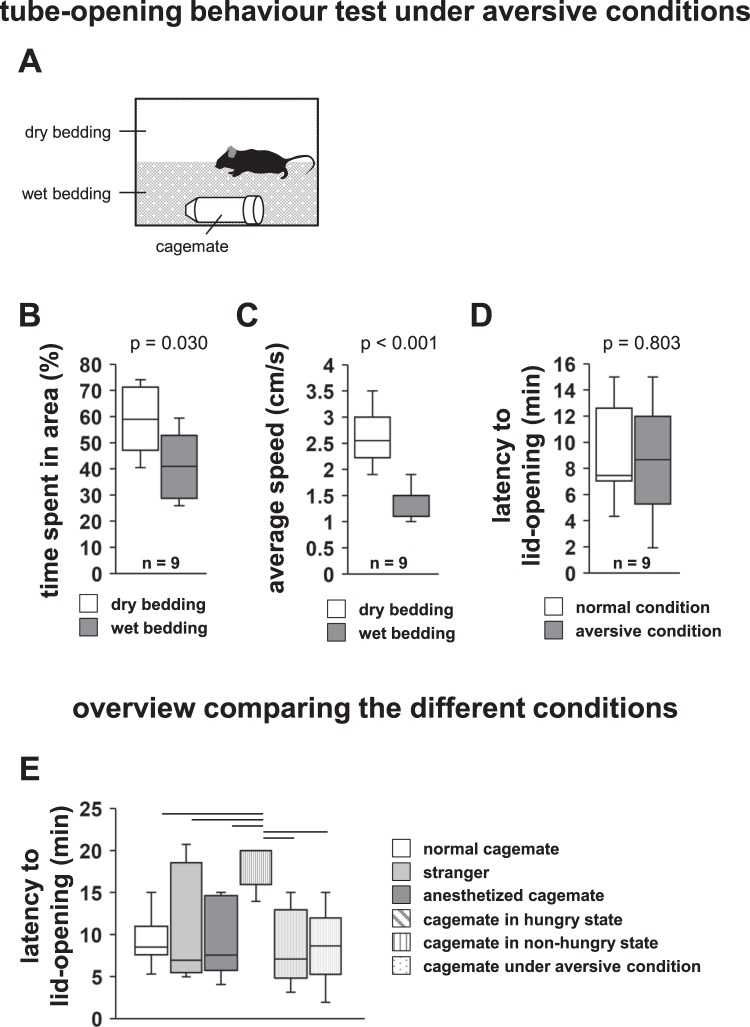
Tube-opening behaviour test under aversive conditions. (**A**) Schematic diagram of this test. Half of the home cage contains wet bedding and the other half contains dry, normal bedding. Tube containing the cagemate mouse on the wet bedding. (**B**) Tube-opening behaviour test for cagemate under the aversive condition: time spent in the area. (**C**) Average speed of the test mouse in each bedding. (**D**) Comparison of the latency to lid-opening when the tube contains the cagemate is placed on wet or normal bedding. (**E**) Latency to lid-opening in various conditions of the tube-opening behaviour test. All data are presented as box plots. (**E**) Statistical significance is represented by top bars: *p < 0.05. The p values were calculated using paired t-test (**B,C**) and one-way repeated measures ANOVA (**D,E**). n = 9 animals per test.

We examined whether the subject mouse would engage in tube-opening behaviour to free constrained cagemate mice even when the tube-opening procedure could not be easily performed. We wet half of the home cage bed, creating an environment where the subject mouse would be reluctant to approach the tube containing the cagemate (Fig. 4A). Subject mice spent less time in the area with the cagemate mouse on the wet bedding than in the empty dry bedding area (Fig. 4B, *t*_16_ = 2.424, p = 0.030). Consistently, subject mice showed reduction in the average speed in the wet bedding area than in the dry bedding area (Fig. 4C, *t*_17_ = −6.219, p < 0.001). We observed that subject mice opened the paper lid of the tube on the wet bedding containing the cagemate. We compared the latency to lid-opening for the cagemate- containing tubes placed on wet and dry bedding (Fig. 4D, *F*_1,16_ = 0.65, p = 0.803). Subject mice showed equivalent latency to lid-opening in both states. These results suggest that subject mice show tube-opening behaviour to free constrained cagemate mice even under aversive conditions.

### Comparison among the different conditions

We compared the latency to lid-opening for the tube containing the cagemate under different conditions (Fig. 4E, *F*_5,33_ = 4.245, p = 0.003). The latency to lid-opening was significantly increased in the hungry state compared to that in the other conditions. There were no significant differences among the other conditions.

### Degree of motivation towards constrained and non-constrained cagemates

**Figure 5:**
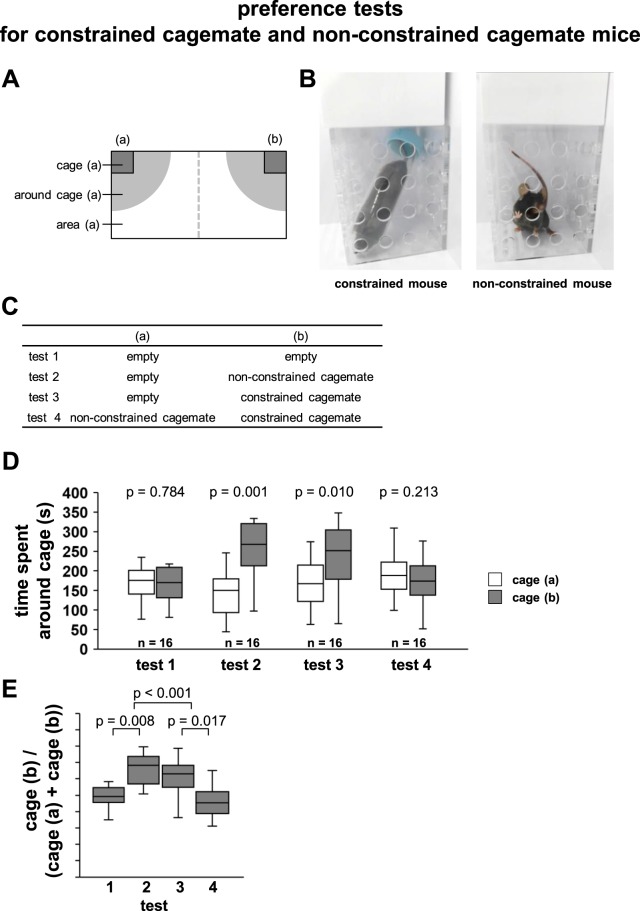
Preference tests for constrained cagemate and non-constrained cagemate mice in the social interaction test apparatus. (**B**) Schematic diagram of the apparatus of this experiment. Two transparent cages [(a), and (b)] are placed at both ends of a rectangular apparatus, and half of the area of the apparatus is taken as the respective area [area (a) and area (b)]. A radius of 20 cm around the transparent cage was set around the cage [around cage (a) and around cage (b)]. (**B**) Sample picture of the transparent cages containing constrained and non-constrained cagemates. (**C**) Test schedule. For each mouse, four tests were conducted according to the contents of the table. Cagemates in each state were placed in transparent cages (a) and (b). Preference tests for constrained cagemate and intact mice: time spent around the cage (**D**), and preference index defined as (time spent around cage (a))/(time spent around cage (a) + time spent around cage (b)). All data are presented as box plots. The p values were calculated using Wilcoxon signed-rank test (**D**) and one-way ANOVA (**E**). n = 16 animals per trial.

We investigated whether the motivation for the helping-like behaviour towards a constrained cagemate would be equivalent to the interest towards a non-constrained cagemate. There were no significant differences in the time spent in each area when both areas were empty (Fig. 5D, T = 61, n = 16, p = 0.784). Subject mice showed a preference for spending time around the cage with the non-constrained cagemate mouse (Fig. 5D, T = 9, n = 16, p = 0.001). Next, constrained cagemate mice were placed in transparent cages. Subject mice showed a preference for spending time around the cage with the constrained cagemate mouse (Fig. 5D, T = 23, n = 16, p = 0.010). Next, both non-constrained and constrained cagemate mice were placed in transparent cages, which were placed at the corners of the chamber. No significant differences were found between the time spent around the cage with the non-constrained cagemate mouse and that around the opposite-positioned cage, with the constrained cagemate mouse (Fig. 5D, T = 43, n = 16, p = 0.213). These results indicate that subject mice showed equal preference for the constrained and non-constrained cagemate mice (Fig. 5E, *F*_*3,62*_ = 7.784, p < 0.001; test 1 vs. test 2: p = 0.008; test 1 vs. test 3: p = 0.217; test 1 vs. test 4: p = 1.000; test 2 vs. test 3: p = 1.0; test 2 vs. test 4: p < 0.001; test 3 vs. test 4: p = 0.017).

## Discussion

In this study, we investigated for the first time whether mice show tube-opening behaviour to free conspecifics.

Mice engaged in tube-opening behaviour to free conspecifics constrained inside tubes. Since the mice did not open the paper lid of empty tubes, their behaviour can be considered to be as motivated by the constrained conspecifics or restraining apparatus, or specific aspects of these. It has already been reported that rats exhibit similar behaviours, by rescuing other rats trapped in tubes or in flooded environments^9–12^. Considering that the mouse is also a rodent, the results of this study are consistent with those of the previous reports.

In the previous studies, the helping behaviour exhibited by rats involved a very simple task of moving a lid to release the trapped conspecific^9–12^. In this experiment, we constructed a more complicated task, in which tube-opening behaviour could only performed with damage to the paper lid, which required time and effort. The latency of opening the paper lid was shortened through repeated trials. Moreover, the act was selectively carried out for the tube containing a conspecific individual. Thus, in our study, the behaviour may not be attributed to chance. In this paradigm, mice had to exert focused effort in chewing or crushing the paper to release their cagemate. This study shows that the mouse performed the tube-opening behaviour with a purpose.

Previous studies have suggested that rats are prosocial and that their helping behaviour was motivated by empathy^9–12^. It has been previously reported that rodents exhibit empathetic behaviour only towards cagemates^9,20–22^. However, in this study, the mice showed tube-opening behaviour both to free cagemates and stranger conspecifics. In addition, all mice were accustomed to the tube environment in our present study, and it can be considered that they were not fearful of the tube and thus could be interested in exploring its contents. Subject mice often exhibited tube-opening behaviour to free conspecifics and then entered the tube. Therefore, the act of opening the tube performed here may be based on other motives such as seeking social contact with the restrained mouse rather than expressing empathy^15–17^ or by interest in the restraining device. Further research is needed to clarify the underlying motivation for engaging in this type of behaviour^23^.

In the present study, even in the absence of expression of distress or vocalisation by the anesthetised mouse in the tube, the subject mouse exhibited tube-opening behaviour. It was previously considered that a voiced warning would be necessary to induce helping-like behaviour^10,24^. Our mouse data contradicts a previous rat study^10^. However, distress may not be signalled solely by alarm calls; most likely, for mice as well as for humans, it is multi-modal, and includes olfactory as well as visual and auditory components. In fact, the anesthetised mice were not in the normal state seen in the home cage. Previous studies have shown that animals have the ability to observe the movement of others and identify their state. Mice are attentive to cagemates that show abnormal behaviours^25,26^. Hyperalgesia in mice observing a mouse in pain depends on the visual input of the conspecific in pain^27^. The present data at least suggest that auditory information was not indispensable for the subject mice to engage in tube-opening behaviour to free conspecifics.

Hungry mice tended to open the paper lid of the tube containing the food before engaging in tube-opening behaviour to free conspecifics. However, the hungry mice opened the paper lid of the tube containing constrained conspecifics within the test time. First, this result clearly shows that the mice recognise objects in the tube using sight or olfaction. Second, it indicates that tube-opening behaviour in mice changes based on internal factors. In previous studies, rats prioritised fellow rescue rather than bait reward^9–12^. This was attributed to the ability to understand and actively respond to the emotional state of conspecifics^9–12^. Our mouse data contradict previous rat studies. However, in these studies, the rats had not been starved. Therefore, the deviation from our results may be attributable to the state of hunger and species differences. It has long been demonstrated that mouse behaviour changes depending on hunger and satiety status. Generally, when mice are subjected to maze tasks, a bait is used as a reward and the animals are tested while hungry^28,29^. Our results suggest that the partner’s emotional state is not a casual factor for the tube-opening behaviour.

Mice engaged in tube-opening behaviour to free conspecifics even at the cost of personal discomfort. A wet floor is considered a stressor and is commonly used in stress paradigms, especially chronic unpredictable stress^30^. We showed that the mice walked slower on the wet bedding, which indicated that the experience was aversive. Nevertheless, the mice did walk on the wet bedding to help their constrained conspecifics. This result shows that there is a strong motivation, purpose, and/or interest in the tube-opening behaviour to free conspecifics.

Social interest in mice can be directly measured through their approach or avoidance behaviour^31,32^. In this study, mice showed a similar degree of motivation towards constrained and freely moving conspecifics, which showed that they neither avoided nor had increased interest towards the constrained mice. Rescue behaviour as a prosocial behaviour is psychologically oriented towards the goal of reducing another’s suffering. However, our results indicate that mice do not fear or show heightened interest towards the constrained state. Therefore, it is possible that the tube-opening behaviour observed here is not rescue or prosocial behaviour.

Our results indicate the possibility that the tube-opening behaviour of the mice may not be oriented towards the rescue of restrained conspecifics from suffering. The mental processes of mice, rats, or apes are unknown and it is difficult to decipher the motivational states of such animals. Whether the underlying motivation for the tube-opening behaviour involves empathy, sociality, or other factors is unknown, and further research is needed. However, our results show that mice can show rescue-like behaviour as do rats. An effective mouse model for rescue-like behaviour research will facilitate the study of aspects of this behaviour, which would otherwise not be possible.

## Conclusion

This study clearly suggests that mice show helping-like behaviour towards conspecifics. The mouse identifies the contents of the restraining tube and engages in tube-opening behaviour to free other mice. This tube-opening behaviour depends on internal factors. It is not clear whether this rescue-like behaviour is prosocial, aimed towards rescuing conspecifics; however, our results clearly showed that engaging in this tube-opening behaviour is strongly motivated.

## Methods

### Animals

All animal experiments were performed in accordance with the U.S. National Institutes of Health (NIH) Guide for the Care and Use of Laboratory Animals (NIH Publication No. 80–23, revised in 1996) and approved by the Committee for Animal Experiments at Kawasaki Medical School Advanced Research Center. All efforts were made to minimise the number of animals used and their suffering. Animals were purchased from Charles River Laboratories (Kanagawa, Japan) and housed in cages (five animals per cage) with food and water provided *ad libitum* under a 12-h light/dark cycle at 23 °C–26 °C. We used C57BL/6N male mice aged 10 weeks. All behavioural tests were conducted in behavioural testing rooms between 08.00 and 18.00 h during the light phase of the circadian cycle. After the tests, all equipment was cleaned with 70% ethanol and super hypochlorous water to prevent bias based on olfactory cues. Behavioural tests were performed according to the test order described below.

### Training for paper-lid opening

Mice were subjected to training for opening the paper lid on being constrained inside 3-cm diameter transparent plastic cylinders (50-mL tubes); for this, the front of the tube was closed with a paper lid and the rear was closed with a plastic lid (Fig. 1A). The tube was placed inside a new home cage, and after the mice managed to break through the paper lid and exit the tube, they were allowed to act freely for 5 min. The exercise was performed three times a day for 2 days. All the mice were able to open the paper lid and exit the tubes.

### Tube-opening behaviour to free cagemate mice

We used 50-mL tubes with a cut tip. We closed the back of the empty tube and of the tubes containing constrained mice with a paper lid (Fig. 1B,C). In this test, a familiar mouse (cagemate) was placed in one of the transparent tubes located at the sides of a new home cage. We placed an empty tube on the other side (Fig. 1D). The constrained cagemate mice had also been trained to open the paper lid. The subject mouse was placed at the centre and allowed to explore the entire home cage for a 90-min session (Fig. 1E). The latency to lid-opening of the tube containing the cagemate mouse was measured. After lid-opening, the mice were allowed to act freely for 5 min. The mice had access to also open the paper lid of the empty tube. We performed the test once a day for 7 days. Seventy percent of the mice learned to open the lid. Mice that did not learn to open the lid were excluded from the analysis. The same subject mice were tested in all conditions. The data were recorded on video.

### Tube-opening behaviour to free cagemate and stranger mice

In this test, a familiar mouse (cagemate) or unfamiliar C57BL/6N male mouse (stranger) that had no previous contact with the subject mouse was placed in one of the transparent tubes located at the sides of a new home cage (Fig. 2A). The subject mouse was placed at the centre and allowed to explore the entire home cage for a 30-min session. The test was terminated when the mouse opened the paper lid. The cagemate area referred to the half of the home cage with the tube containing the cagemate, and the stranger area referred to the half of the home cage with the tube containing the stranger mouse (Fig. 2A). The latency to lid opening and the amount of time spent in each area during the 30-min sessions were measured. The time spent in the area was measured only until one of the tubes was opened. Mice that did not open either of the two tubes were excluded from the analysis. The data were recorded on video and analysed using the ANY-MAZE software.

### Tube-opening behaviour to free anesthetised cagemate mice

The cagemate mice were deeply anesthetised with a high dose of sodium pentobarbital (50 mg/kg, i.p.). The anesthetised mice were placed in transparent tubes that were closed on one side with a paper lid. The anesthetised cagemate area referred to the half of the home cage with the tube containing the anesthetised cagemate mouse, and the empty area referred to the half of the home cage with the empty tube (Fig. 2E). The latency to lid opening and the amount of time spent in each area during the 30-min sessions were measured. The time spent in the area was measured only until the tube was opened. We compared the latency to lid-opening for tubes containing the anesthetised mice and tubes containing unanesthetised mice. The test was terminated when the mouse opened the paper lid. Mice that did not open either of the two tubes were excluded from the analysis. The data were recorded on video and analysed using the ANY-MAZE software.

### Tube-opening behaviour in hungry and non-hungry states

To induce hunger, we restricted access to food from the day before the test. For the test, we used a transparent tube containing food and closed on one side with a paper lid (Fig. 3A). In this test, a tube containing a cagemate and a tube containing food were placed on opposite sides of a new home cage (Fig. 3A,B). The cagemate area referred to the half of the home cage with the tube containing the cagemate, and the feed area referred to the half of the home cage with the tube containing the food (Fig. 3B). The latency to lid opening and the amount of time spent in each area during 30-min sessions were measured. The time spent in the area was measured only until the tube containing the cagemate was opened. The test was terminated when the mouse opened the paper lid. Mice that did not open either of the two tubes were excluded from the analysis. The data were recorded on video and analysed using the ANY-MAZE software. We performed the same experiment using subject mice that were not food deprived (Fig. 3E,F).

### Tube-opening behaviour under aversive conditions

We wet half of the home cage bedding with water. Thus, half of the home cage contained wet bedding and the other half contained dry, normal bedding. A transparent tube containing a cagemate mouse was placed on the wet bedding (Fig. 4A). The latency to lid-opening and the amount of time spent in each area during 30-min sessions were measured. The time spent in the area was measured only until the tube was opened. We also analysed the average speed at which the subject mouse moved in each area. We compared the latency to lid-opening under normal and aversive conditions. The test was terminated when the mouse opened the paper lid. Mice that did not open either of the two tubes were excluded from the analysis. The data were recorded on video and analysed using the ANY-MAZE software.

### Preference tests for constrained and non-constrained cagemate mice using social interaction test apparatus

The apparatus had a rectangular shape (30 × 60 × 40 cm). Two transparent cages (7.5 × 7.5 × 10 cm, with several holes with a diameter of 1 cm) [(a), and (b)] were placed at both ends of the rectangular apparatus (Fig. 5A,B). Each mouse was placed in the box for 10 min and allowed free exploration for habituation. In this test, we tested each mouse according to the table schedule (Fig. 5C). In test 1, both cage (a) and cage (b) were empty. In test 2, cage (a) was empty and a non-constrained cagemate was placed inside cage (b). In test 3, a constrained cagemate was placed inside cage (a) and cage (b) was empty. In test 4, a constrained cagemate was placed inside cage (a) and a non-constrained cagemate was placed inside cage (b). The non-constrained cagemate mouse was placed inside the transparent cage, which allowed nose contact between the bars but prevented the mice from fighting with each other. The subject mouse was placed at the centre and allowed to explore the entire box for a 10-min session. One side of the rectangular area was identified as the cage (a) area and the other as the cage (b) area. The amount of time spent in each area and around each cage during 10-min sessions was measured. We also analysed the average speed at which each subject mouse moved in each trial. The apparatus was cleaned after each phase of this test. In this test we used naive mice, not used in other tests. The data were recorded on video and analysed using the ANY-MAZE software.

### Statistical analyses

Statistical analysis was conducted using the SPSS software (IBM Corp, Tokyo, Japan). If the variables were found to be non-normally distributed, non-parametric analysis was used. We used the non-parametric Mann-Whitney *U*-test, Wilcoxon signed-rank test, Student’s *t*-test, paired *t*-test, one-way ANOVA, or one-way repeated measures ANOVA. A p value < 0.05 was regarded as statistically significant. Data are shown as box plots.

## Supplementary information

Supplementary Figure Legends

Supplementary Video 1

Supplementary Video 2
