# Supplementary material for: Helping-Like Behaviour in Mice Towards Conspecifics Constrained Inside Tubes
Source: Sci Rep. 2019 Apr 9;9:5817. doi: 10.1038/s41598-019-42290-y (PMC6456590; doi:10.1038/s41598-019-42290-y)
Supplement: Supplementary file 1 — Supplementary Figure Legends [file 41598_2019_42290_MOESM1_ESM.docx]

1. ORIGINAL RESEARCH ARTICLE

**Helping-Like Behaviour in Mice** **Towards Conspecifics Constrained Inside Tubes**

**Hiroshi Ueno^1,2,^*, Shunsuke Suemitsu^3^, Shinji Murakami^3^, Naoya Kitamura^3^,　Kenta Wani^3^, Yosuke Matsumoto^4^, Motoi Okamoto^2^, Takeshi Ishihara^3^**

1. 1 Department of Medical Technology, Kawasaki University of Medical Welfare, Okayama, 701-0193, Japan
2. 2 Department of Medical Technology, Graduate School of Health Sciences, Okayama University, Okayama, 700-8558, Japan
3. 3 Department of Psychiatry, Kawasaki Medical School, Kurashiki, 701-0192, Japan
4. 4 Department of Neuropsychiatry, Graduate School of Medicine, Dentistry and Pharmaceutical Sciences, Okayama University, Okayama, 700-8558, Japan

**Author Information**

Hiroshi Ueno, PhD. E-mail: dhe422007@s.okayama-u.ac.jp

Shunsuke Suemitsu, PhD, MD. E-mail: ssue@med.kawasaki-m.ac.jp

Shinji Murakami, PhD, MD. E-mail: muraka@med.kawasaki-m.ac.jp

Naoya Kitamura, PhD, MD. E-mail: n-kitamura@med.kawasaki-m.ac.jp

Kenta Wani, PhD, MD. E-mail: kenta99101@yahoo.co.jp

Yosuke Matsumoto, PhD, MD. E-mail: ymatsumoto@cc.okayama-u.ac.jp

Motoi Okamoto, PhD, MD. E-mail: mokamoto@md.okayama-u.ac.jp

Takeshi Ishihara, PhD, MD. E-mail: t-ishihara@med.kawasaki-m.ac.jp

*Corresponding author.

1. Hiroshi Ueno, PhD.
2. Address: Department of Medical Technology, Kawasaki University of Medical Welfare, 288, Matsushima, Kurashiki, Okayama, 701-0193, Japan
3. Phone: +81-86-462-1111, Fax: +81-86-462-1193
4. E-mail address: dhe422007@s.okayama-u.ac.jp (H. Ueno)

**Supplementary Figure Legends**

**Supplementary Video 1** Helping behaviour towards a cage-mate mouse in the home cage.

It is a 4 × speed movie. Three trial images.

**Supplementary Video 2** Helping behaviour towards an anesthetised cage mate in the home cage.

It is a 4 × speed movie. Two trial images.
